# Systematic analysis of biological roles of charged amino acid residues located throughout the structured inner wall of a virus capsid

**DOI:** 10.1038/s41598-018-27749-8

**Published:** 2018-06-22

**Authors:** Pablo J. P. Carrillo, Marta Hervás, Alicia Rodríguez-Huete, Rebeca Pérez, Mauricio G. Mateu

**Affiliations:** 1 Centro de Biología Molecular “Severo Ochoa” (CSIC-UAM), Universidad Autónoma de Madrid, Cantoblanco, 28049 Madrid, Spain; 2 Present Address: Centro Nacional de Biotecnología, Campus de la Universidad Autónoma de Madrid, 28049 Madrid, Spain; 3 Present Address: Departamento de Ingeniería Química y Tecnología del Medio Ambiente, Universidad de Valladolid, 47011 Valladolid, Spain

## Abstract

Structure-based mutational analysis of viruses is providing many insights into the relationship between structure and biological function of macromolecular complexes. We have systematically investigated the individual biological roles of charged residues located throughout the structured capsid inner wall (outside disordered peptide segments) of a model spherical virus, the minute virus of mice (MVM). The functional effects of point mutations that altered the electrical charge at 16 different positions at the capsid inner wall were analyzed. The results revealed that MVM capsid self-assembly is rather tolerant to point mutations that alter the number and distribution of charged residues at the capsid inner wall. However, mutations that either increased or decreased the number of positive charges around capsid-bound DNA segments reduced the thermal resistance of the virion. Moreover, mutations that either removed or changed the positions of negatively charged carboxylates in rings of acidic residues around capsid pores were deleterious by precluding a capsid conformational transition associated to through-pore translocation events. The results suggest that number, distribution and specific position of electrically charged residues across the inner wall of a spherical virus may have been selected through evolution as a compromise between several different biological requirements.

## Introduction

Viruses provide excellent model systems to investigate relationships between atomic structure, physicochemical properties and biological function of biomacromolecular complexes^1–4^. One important finding of these studies is that some specific, electrically charged groups in virus particles play a biological role. In particular, attractive ionic interactions between structural proteins may be required during the infectious cycle. For example, metal ion-mediated carboxylate cages stabilize the native conformation of cowpea chlorotic mottle virus (CCMV)^5^; a cluster of charges in the Rous sarcoma virus capsid is important for assembly and maturation^6^; ionic interactions between the infectious bursal disease virus capsid and scaffolding proteins contribute to regulate assembly^7^. In turn, either permanent or transient electrostatic repulsions between capsid subunits may limit virus stability and facilitate biologically required conformational transitions, disassembly and/or uncoating in different viruses (e.g., tobacco mosaic virus^8,9^, CCMV^5,10,11^, foot-and-mouth disease virus^12,13^, human immunodeficiency virus^14–17^ and simian virus 40 (SV40))^18^.

Electrostatic interactions between capsid and nucleic acid may also play a biological role in viruses^5,19–34^. In particular, positively charged residues in structural proteins may stabilize the virion by neutralizing the excess negative charge of the viral nucleic acid phosphates that is not neutralized through interactions with metallic and/or organic (poly)cations^26,35–37^. In double stranded (ds) DNA viruses (e.g., SV40^21^ and adenovirus^32^) neutralization of nucleic acid charge is partly achieved by basic viral proteins inside the virus particle. In many icosahedral single stranded (ss) RNA viruses, positively charged residues clustered in disordered terminal segments of capsid protein subunits neutralize a large part of the RNA charge^5,19,23–25,27,30,33^. Charge neutralization promotes virus morphogenesis by facilitating the packaging of dsDNA into preformed capsids^26^ or the coassembly of ssRNA with capsid proteins, as revealed by experiment and justified in physicochemical terms by theoretical studies^34,38–48^. Repulsive interactions between capsid and viral nucleic acid can also be biologically relevant. For example, in phage HK97 charge-charge repulsion between dsDNA being packaged and the capsid inner wall may facilitate a conformational transition during virion maturation^22^.

The experimental studies referred to above have focused on a few specific charged groups in the viral particle. To our knowledge, no experimental study has systematically investigated for any spherical virus the roles of most of the many charged residues located throughout the structured capsid inner wall (outside disordered peptide segments). In the present study we have addressed this question using as a model one of the smallest and structurally simplest nonenveloped icosahedral viruses known, the minute virus of mice (MVM).

**Figure 1:**
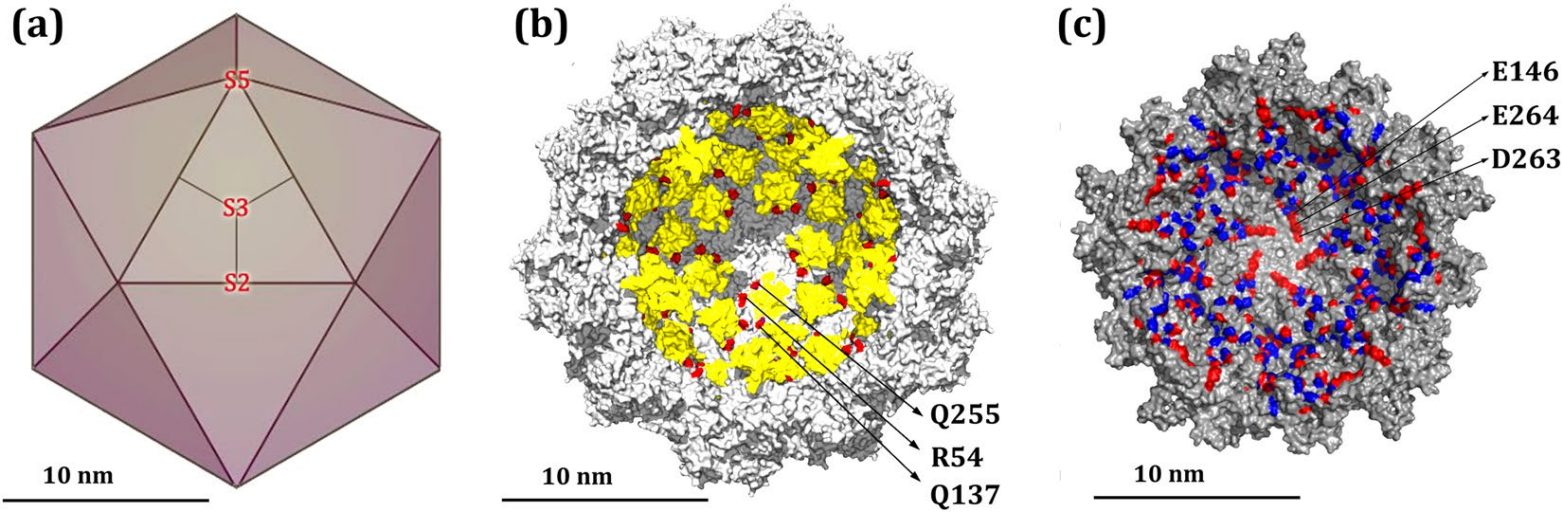
Structure of the MVM capsid and distribution of capsid-bound DNA segments and electrically charged residues at the capsid inner wall. (**a**) Scheme of the icosahedral MVM capsid architecture. Trimeric CBBs are idealized as triangles and the interfaces between the three capsid subunits in one trimer (center) are idealized as straight lines. S5, S3 and S2 symmetry axes are indicated. (**b**) Cross-section of the atomic structure of the MVM virion^51,52^. Structurally equivalent ssDNA segments bound to equivalent sites at the capsid inner wall are colored yellow. Residues R54, Q137 and Q255 close to the capsid-bound DNA segments are colored red, and those surrounding one DNA segment are labelled. (**c**) Distribution of electrically charged residues at the structured inner wall of the MVMp capsid^52^. For clarity, only a pentamer of trimers around a S5 axis (15 VP2 subunits, one fourth of the capsid) is represented, as seen from the capsid interior. Basic or acidic residues are respectively coloured blue or red. Residues E146, D263, E264 of five capsid subunits (labelled for one subunit) define a conspicuous ring of 15 negatively charged carboxylates that surrounds each capsid pore (center).

Viruses of the *Parvoviridae* family including MVM^49,50^ show excellent potential for oncolysis, gene therapy and bio/nanotechnological applications, which has contributed to promote intensive research on these viruses. The atomic structures of MVM virion and empty capsid have been determined by X-ray crystallography^51,52^ (Fig. 1). The icosahedral T = 1 capsid (Fig. 1a) is formed by 60 subunits (VPs) with identical fold and sequence, except for their disordered N-terminal segments (Nt). VP1 is produced by alternative splicing from the VP1/VP2 gene, differs from VP2 by having a longer Nt, and contributes about 10 of the 60 capsid subunits; VP2, the fundamental capsid protein, is able to self-assemble into VP2-only capsids that are structurally indistinguishable from VP1/VP2 capsids^52^, except for the absence of the VP1 Nts in the capsid interior; VP3 is a shortened form of VP2 that arises by proteolytic removal of the Nts of some VP2 subunits as the virion initiates infection^50^.

For MVM, trimers of VP subunits constitute stable capsid building blocks (CBBs)^53,54^ that are translocated into the cell nucleus, where capsids are self-assembled from them^53–56^. After the empty capsid has been assembled, the viral ssDNA genome is packaged through one of the pores located at 5-fold symmetry (S5) axes^50,57^. In the virion thus formed, structured segments of the packaged ssDNA are noncovalently bound to equivalent, specific sites at the capsid inner wall^51,52^ (Fig. 1b). Interestingly, analysis of capsid-ssDNA interactions in MVM revealed very few short- or medium-range ionic interactions between DNA phosphates and basic capsid residues^52,58^.

Some biologically relevant, short motifs rich in charged residues have been identified in the disordered VP1 and VP2 Nts which in newly assembled capsids are internally located, but become externalized as the infectious cycle progresses^50,51^. These motifs act as virus trafficking domains and include: (i) two highly basic segments in VP1 Nt (NLS) that function as signals for translocation of both infecting virions and VP1/VP2 trimers into the cell nucleus^53–56^; (ii) two other basic segments in VP1 Nt that, based on sequence homology, were proposed to interact with ssDNA^56^; (iii) several phosphorylated residues grouped in the Nt of some VP2 subunits, with a role in nuclear exit of progeny virions^59,60^ and in initiation of infection^61^. In addition, basic structured motifs in VPs (NLM)^62^ and some phosphorylated VP residues participate in nuclear translocation of CBBs^54–56^.

In this study we have focused on the thus far unknown roles of many of the individual charged amino acid residues located at the structured inner wall of the MVM capsid, outside the Nts and trafficking motifs. As these charged residues may participate in intracapsid and/or capsid-ssDNA ionic interactions in the viral particle, an extensive mutational analysis was carried out to explore their possible role in capsid assembly and/or virus infectivity or stability against thermal inactivation.

## Results

### Number and distribution of electrically charged amino acid residues at the capsid inner wall

The crystal structure of the MVM (strain p) capsid (PDB ID: 1Z14)^52^ was inspected to determine the number and distribution at neutral pH of negatively charged carboxylates and positively charged amino, guanidinium and imidazole groups. Imidazoles were assumed to be positively charged most of the time because the pK_a_ of this group in free histidine (pK_a_~6.8) may be substantially raised in the viral capsid due to the presence of spatially close, negatively charged carboxylate groups^63,64^. The counting of charged amino acid residues was carried out for the natural MVM capsid containing 50 copies of VP2 and 10 copies of VP1. All charged residues in the disordered Nts inside the viral particle are most probably exposed to solvent and are, thus, assumed to belong to the capsid inner surface (until they are externalized during the viral cycle). However, they are loosely connected with the rest of the capsid and do not form a part of the structurally defined, quasispherical capsid inner wall, which is the subject of the present study.

The disordered Nt of each of the 10 VP1 subunits contains 13 negatively charged and 26 positively charged side chains plus the terminal amino group, yielding a positive net charge of +14 per VP1 subunit. This excess positive charge is mainly located in motifs involved in nuclear translocation. The disordered Nt of each of the 50 VP2 subunits contains 4 negatively charged and 3 positively charged side chains plus the positively charged terminal amino group, yielding a net charge of 0. The structured inner wall in the MVM capsid contains 14 negatively charged and 14 positively charged side chains in each of the 60 capsid subunits, again yielding a net charge of 0.

In total, if post-translational modifications (phosphorylation) were disregarded, the MVMp capsid inner surface, including the Nts, would contain 1170 negatively charged and 1310 positively charged groups, with the modest excess positive charge (+140) being due to the VP1 Nts. In fact, the presence of an undefined number of phosphorylated residues in the capsid interior (e.g., in VP2 Nts^59,60^) results in a capsid inner surface with a weakly negative net charge, depending on the number of subunits in which different residues are phosphorylated.

The spatial distribution of charged groups in the structured capsid inner wall (i.e., excluding the disordered Nts) is represented in Fig. 1c. In general, charge distribution is rather homogeneous, with most of the negatively charged groups located in close proximity to the positively charged groups and *vice versa*, which contributes to mutual charge neutralization. However, some regions in the capsid inner wall show a non-neutral charge distribution. In particular conspicuous rings, each made of 15 negatively charged residues, were detected around and relatively close to the pores at capsid S5 axes (Fig. 1c). These rings are formed by residues E146, D263 and D264 of each of the S5-related capsid subunits.

The same analysis was carried out for MVM strain i (PDB ID: 1Z1C)^52^. The number of charged residues at the capsid inner surface and their distribution in MVMi and MVMp are similar. Furthermore, sequence comparisons revealed that many charged residues in the capsid inner wall are remarkably conserved among parvoviruses evolutionarily related to MVM, including viruses whose sequence identity in the VP2 capsid protein was only ≤50%^65^ (Table 1 and data not shown). The high degree of conservation of those charged residues suggested they could be functionally important.

**Table 1:** Effects of mutations at the structured capsid inner wall on capsid assembly, virus infectivity and virion resistance against thermal inactivation.

Group	Mutation	Interactions lost^a^			Conservation^b^	Assembly efficiency ratio (%)^c^	Infectivity ratio (%)^d^	Thermal inactivation ratio *k*_*off*_^e^
		Salt bridges	Hydrogen bonds	van der Waals contacts				
	wt					100	100	1
1	R54A	1(E62)			5	91 ± 9*	7 ± 10*	1.87 ± 0.5*
	K471A			2(2)	7	69 ± 18*	10 ± 10*	1.03 ± 0.14
	K478A		2(L475)	5(1)	7	94 ± 4	1 ± 0.5*	ND
	R480A		4(H477,K478,Y450)	28(9)	6	112 ± 6*	64 ± 40	0.62 ± 0.42
	K490A		1(N275)	4(1)	4	68 ± 6*	36 ± 80	1.08 ± 0.5
2	D115A		3(N117,A191)	8(3)	7	5 ± 1*	<threshold*	NA^g^
	E146A			4(3)	7	118 ± 2*	<threshold*	NA
	D263A	1(R260)	1(S43)	10(3)	7	100 ± 7*	<threshold*	NA
	E264A			1(1)	6	106 ± 1*	<threshold*	NA
	E472A	2(H482)		5(3)	6	101 ± 24	19 ± 8*	1.26 ± 0.16*
	D474A		1(K278)	6(0)	7	63 ± 24*	<threshold*	NA
3	Q137K			2(0)	1	116 ± 18	145 ± 68	2.66 ± 0.04*
	S182H				5	91 ± 1*	<threshold*	NA
	Q255R			3(0)	1	131 ± 10*	88 ± 34	2.27 ± 0.22*
	T257K				2	111 ± 4*	69 ± 56	1.34 ± 0.02*
	N275K		1(L490)		1	116 ± 12*	113 ± 69	1.02 ± 0.23
4	E146Q				7	ND^f^	35 ± 27*	ND
	E146D				7	ND	77 ± 17*	ND
	D263N				7	ND	0.13 ± 0.17*	ND
	D263E				7	ND	0.077 ± 0.076*	ND
	E264Q				6	ND	0.0037 ± 0.0016*	ND
	E264D				6	ND	0.035 ± 0.014*	ND
	E146Q/D263N/E264Q				7/7/6	ND	<threshold*	NA
	E146D/D263E/E264D				7/7/6	ND	0.1 ± 0.008*	ND

^a^The number of intracapsid noncovalent interactions of different types (salt bridges, hydrogen bonds, van der Waals contacts) lost by mutation of the specified residue to alanine (number, type and, in parenthesis, other residues involved) are indicated. For van der Waals (vdW) contacts, two numbers are given: total number of vdW contacts lost and (in parenthesis) number of vdW contacts between carbon atoms (“hydrophobic” contacts). The cutoff distances chosen to define interactions are as given in ref.^66^.

^b^The degree of conservation of the specified residue among MVM and six other parvoviruses closely related to MVM is indicated by the number of these parvoviruses (1 to 7) in which that residue is present. Parvoviruses compared and (in parenthesis) percent identity in the VP2 capsid protein relative to MVM are: Hamster H1 Parvovirus (68%); Raccoon Parvovirus (52%); Canine Parvovirus (52%); Feline Parvovirus (52%); Porcine Parvovirus (50%), Mink Aleutian Parvovirus (35%)^65^.

^c^Assembly efficiency of each mutant capsid relative to the wt, obtained in *in situ* immunofluorescence experiments. In each case, many cells were visualized, and the number of cells that yielded a positive signal when anti-capsid antibody was divided by the number of cells that yielded a positive signal when anti-capsid protein antibody was used (Fig. 2a). For each mutant, average assembly efficiency was determined from counting cells in 15–25 different fields in each of two independent experiments. The assembly efficiency ratio is expressed as a percentage: (assembly efficiency of mutant capsid/assembly efficiency of wt capsid) × 100. Average values ± standard deviations (SD) are given. Differences in average values relative to wt that corresponded to ≥1 standard deviation were taken as statistically significant (with 66% confidence) and are indicated with an asterisk.

^d^Infectious titer of each mutant virus relative to the wt, obtained in transfection experiments. For each mutant, the average infectious titer was determined from values obtained in two independent experiments, each performed in duplicate (4 determinations in each case). The infectivity ratio is expressed as a percentage (mutant titer/wt titer) × 100. Average values ± standard deviations (SD) are given. Differences in average values relative to wt that corresponded to ≥1 standard deviation were taken as statistically significant (with 66% confidence) and are indicated with an asterisk.

^e^Thermal inactivation rate constant *k*_*off*_ of each mutant virion relative to the wt, obtained in thermal inactivation experiments at 70 °C. For each mutant in each experiment, data obtained at different times were fitted to an exponential decay (Fig. 3a). For each mutant, the average inactivation rate was determined from values obtained in two or three experiments. The inactivation rate ratio is expressed as a percentage (*k*_*off*mutant_/*k*_*off*wt_) ×100. Average values ± standard deviations (SD) are given. Differences in average values relative to wt that corresponded to ≥1 standard deviation were taken as statistically significant (with 66% confidence) and are indicated with an asterisk.

^f^ND, not determined.

^g^NA, not applicable.

### Selection of amino acid replacements for analyzing the effects of altering number and distribution of electrically charged residues at the capsid inner wall

As described above, the inner surface of this ssDNA virus capsid lacks the large excess positive charge found at the inner surface of many ssRNA virus capsids, and shows a peculiar charge distribution: few basic groups close to the capsid-bound ssDNA segments, and conspicuous rings of acidic groups around the capsid pores. We wondered whether these charge-related features of MVM could be required for capsid assembly, virion infectivity and/or virion stability against inactivation.

We started by designing different individual mutations in the MVMp capsid inner wall that: (i) decrease the positive charge (by 60 units) in different capsid regions, by removing amino or guanidinium groups through mutation of specific Lys or Arg residues to Ala (Table 1, Group 1); or (ii) decrease the negative charge (by 60 units) in different capsid regions, by removing carboxylates through mutation of specific Asp or Glu residues to Ala (Table 1, Group 2); or (iii) both increase the positive charge of the capsid inner wall close to capsid-bound ssDNA segments and (presumably) establish short- or medium-range ionic interactions between the capsid and these ssDNA segments, through individual replacement of neutral amino acid residues by basic residues (Table 1, Group 3).

Eleven positively or negatively charged amino acid residues to be mutated to Ala (Table 1, Groups 1 and 2 respectively) were chosen among those more conserved in MVM and related parvoviruses, and with the charged group exposed to solvent on the capsid inner surface. Five polar, electrically neutral residues to be mutated to positively charged residues (Table 1, Group 3) were chosen among those deemed non-critical for viral function: they are generally not conserved among parvoviruses, and have a solvent-exposed side chain that establishes no or few intracapsid interactions, and no interactions with capsid-bound ssDNA segments. In total, 16 residues located at the structured inner wall of each MVMp capsid subunit were chosen for mutational analysis (Table 1, Groups 1–3).

### Functional effects of individually removing or introducing electrically charged groups at the capsid inner wall

#### Effects on capsid assembly

During coassembly of capsid and viral nucleic acid in ssRNA viruses, the electrostatic attraction between capsid subunits with a net positive charge at the inner surface and the negatively charged nucleic acid help overcome any repulsion between equally charged capsid subunits. In contrast, the MVM capsid is assembled in the absence of viral nucleic acid, which is packaged only after the capsid has been formed. Thus, we considered the possibility that the close to zero net charge, and/or the distribution of charged residues at the MVM capsid inner wall, could facilitate self-assembly by minimizing electrostatic repulsion between capsid subunits.

**Figure 2:**
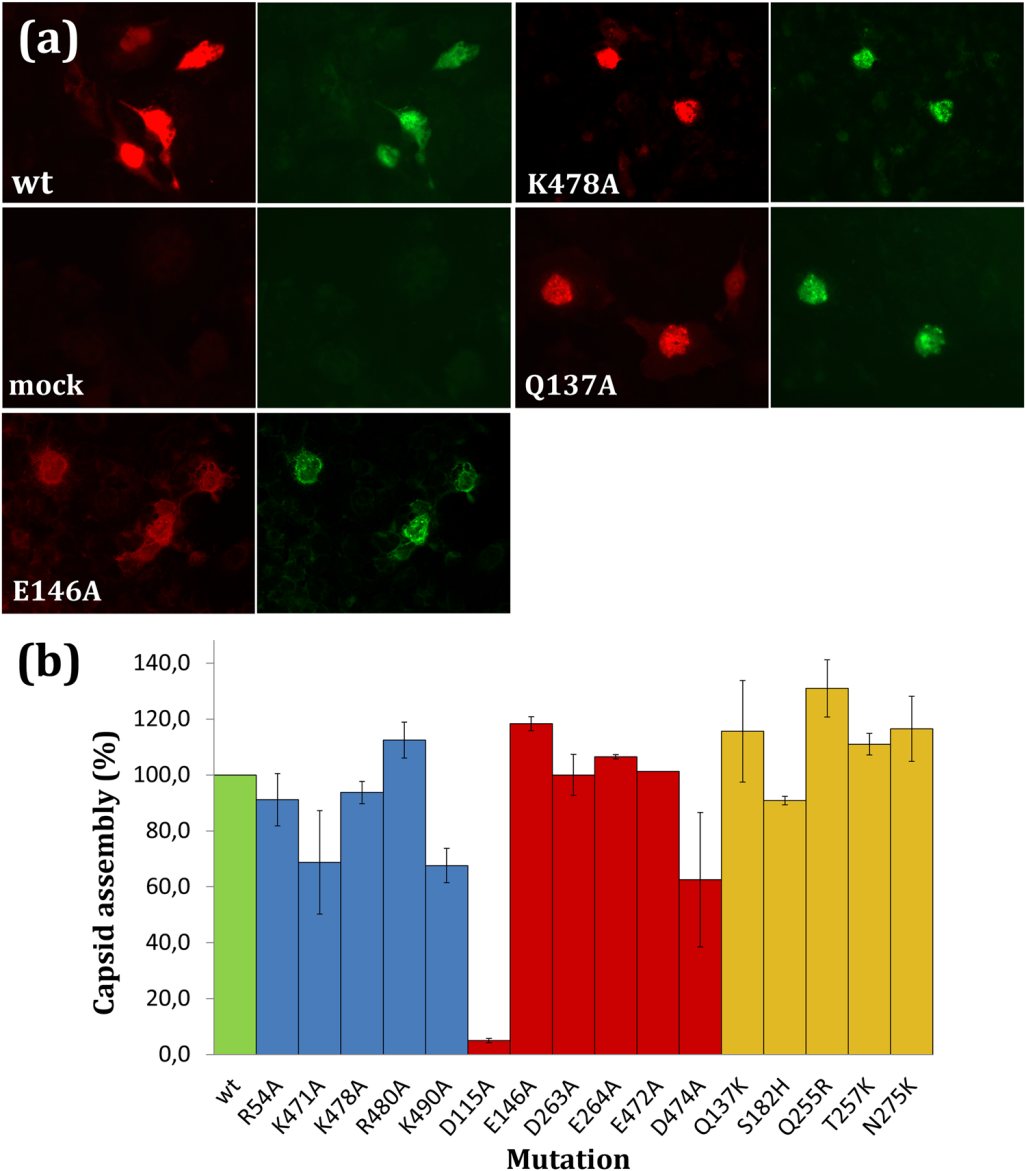
*In situ* immunofluorescence analysis of capsid proteins and capsids produced in mammalian cells transfected with MVM pSVtk-VP1/VP2 plasmids. (**a**) Representative *in situ* immunofluorescence image pairs are shown for cells transfected with wt or representative mutants of Groups 1 (E146A), 2 (K478A) or 3 (Q137K), and for mock-transfected cells as a negative control. In each image pair, the left image corresponds to capsid protein (red fluorescence) and the right image to assembled capsids (green fluorescence). The amounts and avidity of sera and labelled secondary antibodies used to detect either protein or assembled capsid were different, so comparison between signals obtained with different antibodies is not valid. (**b**) Assembly efficiency for each mutant capsid relative to the wt capsid, for which a reference value of 1 has been asigned (green bar). These values were obtained as previously described^55,81^. Mutant plasmids and the wt control plasmid were transfected in parallel using the same batch of cells in a same experiment. Assembly efficiency was determined as described in footnote *c* of Table 1: A large enough number of cells was visualized; the number of those cells that yielded a positive signal (above a sensitivity threshold) when an anti-capsid antibody (green fluorescence) was used was divided by the number of cells that yielded a positive signal (above a sensitivity threshold) when an anti-capsid protein antibody (red fluorescence) was used; and the values obtained for each mutant were normalized. For example: if for a given mutant capsid 80 cells showed green fluorescence and 200 cells showed red fluorescence above a predefined threshold, the absolute assembly efficiency of that mutant capsid was taken as (80/200) × 100 = 40%; if for the for the wt capsid in the same experiment 160 cells showed green fluorescence and 200 cells showed red fluorescence, the absolute assembly efficiency of the wt capsid was (160/200) × 100 = 80%. In this example, the relative assembly efficiency of the mutant capsid compared to the wt capsid would be (40/80) × 100 = 50% Average values were obtained by counting cells in 15–25 fields in each of two independent experiments. Values for mutants of Groups 1, 2, or 3 are respectively indicated by blue, red or yellow bars. Error bars indicate standard deviations (SD). Differences in average values relative to wt that corresponded to ≥1 standard deviation were taken as statistically significant (with a 66% confidence; Table 1).

To analyze this possibility we engineered 16 selected MVM mutant capsids with altered number and distribution of charged groups (see above and Table 1). These mutations were individually introduced in a recombinant plasmid that contains the MVMp capsid protein (VP1/VP2) coding region, and equal amounts of wt and mutant plasmids were used to transfect susceptible cells. The expression of capsid protein and the assembly of empty capsids in transfected cells were analyzed in *in situ* immunofluorescence assays as described in Materials and Methods. The results are shown in Fig. 2 and Table 1. Use of a VP-specific polyclonal antibody showed that all 16 mutants expressed smilar amounts of capsid protein, revealing that VP production was not significantly impaired by any mutation. Use of a capsid-specific monoclonal antibody showed that most (twelve) of these 16 mutations did not impair capsid assembly efficiency (amount obtained were between 90% and 130% that obtained with the wt control in the same experiment). Mutations K471A, K490A and D474A led to moderately reduced yields (60–70% of the wt yield), and only one mutation, D115A, severely inhibited capsid assembly in host cells (∼5% of the wt yield) (Fig. 2).

To sum up, in most tested cases elimination or introduction of electrically charged groups associated with a substantial net charge variation at the capsid inner wall (−60 or +60 units starting with a weak net charge) had no substantial effect on capsid assembly efficiency. Also, most tested, highly conserved, either positively or negatively charged groups at widely different positions in the MVM capsid inner wall were not required for (close to) normal capsid assembly efficiency in a host cell.

#### Effects on virus infection

We considered then that the conserved presence and distribution of charged residues at the capsid inner wall could be required only after the capsid is assembled, during some other step of the viral cycle. For example, it could contribute to a proper electrostatic interaction between capsid and viral nucleic acid during or after genome packaging. Thus, we tested whether any of the 16 mutations that altered the number and distribution of charged groups (Table 1, Groups 1, 2 or 3) had any effect on virus infectivity.

These mutations were introduced in an infectious plasmid containing the MVMp genome, and equal amounts of wt and mutant plasmids were used to transfect host cells. As a control we confirmed, using a VP-specific polyclonal antibody in western blot assays, that no mutation had a discernible effect on VP expression in transfected cells. Then, infectious virion yields were determined for the wt and each mutant in titration experiments carried out in duplicate. The absolute titer obtained for each mutant was normalized relative to the reference titer obtained for the wt virus included as a control in the same experiment. The results obtained with mutants of different groups were different (Table 1, compare Groups 1, 2 and 3).

Firstly, introduction of positively charged groups close to the capsid-bound ssDNA segments had no significant effect on virus yield in all but one of the 5 cases analyzed (Table 1, Group 3). S182H, the only one of these 5 mutations that affected a relatively conserved residue in MVM and other parvoviruses (Table 1), abolished infection. In turn, removal of positively charged groups had no significant effect on virus yield in 2 cases and led to moderate reductions in virus yields (∼1–2 orders of magnitude) in the 3 other cases analyzed (Table 1, Group 1).

In sharp contrast with Group 1 or 3 residues, removal of negatively charged groups, including E146, D263 and E264 at the conspicuous acidic rings surrounding capsid pores, abolished infection in all but one of the 6 cases analyzed (titers below the detection threshold level) (Table 1, Group 2). The exception was E472A, which showed a moderate reduction in infectivity (∼1 order of magnitude).

To sum up, elimination or introduction of positively charged groups at widely different locations in the capsid structured inner wall, with associated net charge variations of −60 or +60, led in most cases to no or only moderate reductions of infectivity. In contrast, removal of negatively charged groups, including those located in conspicuous rings around the capsid pores, generally abolished infectivity.

#### Effects on virion resistance against thermal inactivation

In a previous study we had shown that non-covalent, non-ionic interactions between the MVM capsid inner wall and capsid-bound ssDNA segments stabilize the virion against thermal inactivation of its infectivity^58^ (Fig. 1b). Thus, we considered the possibility that those mutations in Groups 1, 2 or 3 that had no or only moderate effects on infectivity, could still have some effect on virion resistance against thermal inactivation by altering capsid-ssDNA electrostatic interactions.

**Figure 3:**
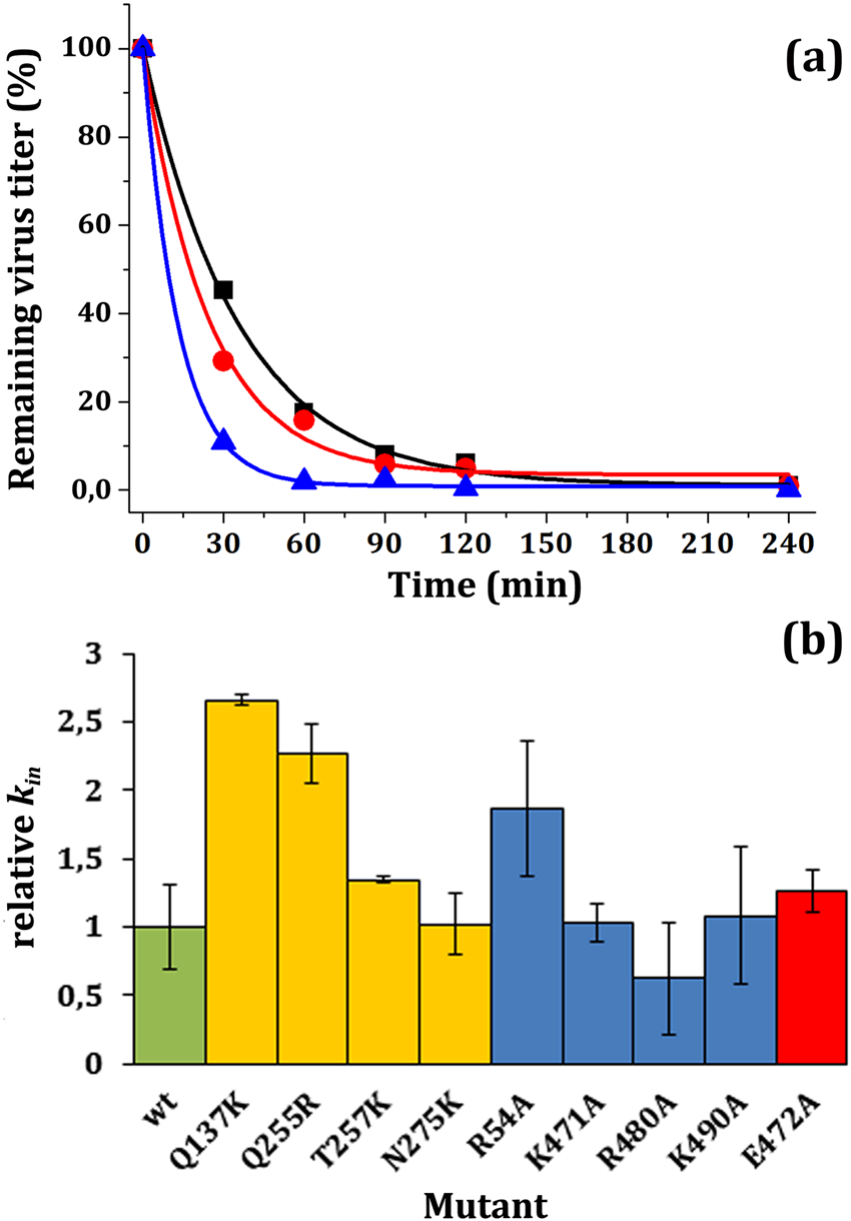
Thermal inactivation of MVM virions. (**a**) Thermal inactivation kinetics of wt virion (black squares) and representative mutant virions T257K (red circles) and Q137K (blue triangles) in a representative experiment at 70 °C.Virus inactivation curves were fitted to exponential decays. Because initial absolute virus titers are very high (in the order of 10^7^ plaque-forming units/ml), virus titers can be determined with similar accuracy over a range of 4-5 orders of magnitude at least. Even at the longest times tested, absolute titers were above 10^3^ plaque-forming units/ml. Thus, titers obtained at every time point were equally accurate and significant in the fitting process to determine the inactivation rate constant, which yielded reasonably low fitting errors and high correlation coefficients (Table 1). (**b**) Relative thermal inactivation rate constants for every tested mutant virion, normalized with respect to the wt rate constant (green bar). Average values obtained for mutants of Groups 1, 2, or 3 are respectively indicated by blue, red or yellow bars. For each mutant, the average inactivation rate was determined from values obtained in two or three experiments. Error bars indicate standard deviations (SD). Differences in average values relative to wt that correspond to ≥1 standard deviation were taken as statistically significant (with a 66% confidence; Table 1).

To test this possibility, 9 infectious mutant virions of Groups 1, 2 or 3 were incubated at 70 °C, and their remaining infectivity was determined as a function of incubation time in two independent experiments, that included equal infectious titers of the wt virion as an internal control (Fig. 3). Thermal inactivation kinetics of wt and mutants followed single exponential decays (see Fig. 3a for representative examples), for which inactivation rate constants were determined. The average rate constants obtained for each mutant were then normalized relative to the wt rate constant (Fig. 3b). The results revealed that 5 out of these 9 mutations had an insignificant effect or, at most, led to a minor reduction in virion resistance against thermal inactivation. The moderately increased resistance against inactivation by mutation R480A was not considered significant according to the criterium used (Table 1) In contrast, mutations R54A, Q137K and Q255R, located close to the capsid-bound DNA segments in the virion (Fig. 1b), significantly reduced the resistance of the MVM virion against thermal inactivation.

### Contribution of negatively charged carboxyates to the preservation of virus infectivity by rings of acidic residues surrounding the capsid pores

We asked next whether the lethal effect of truncating negatively charged side chains at a ring of 15 acidic residues (E146, D263, E264 of five S5-related subunits) around each capsid pore could specifically be due to charge removal. To address this question we produced a new series of mutant capsids (Table 1, Group 4) with different single or multiple mutations at the rings of acidic residues, including: (i) charged to neutral isosteric mutations (carboxylate to amide) that removed the negative charge with minimal steric change; and (ii) Glu to Asp or Asp to Glu mutations that preserved the carboxylate group and its negative charge, but introduced changes in side chain stereochemistry, carboxylate position and, presumably, interactions with neighboring residues in the capsid.

Mutations E146Q and E146D had no or only minor effects on infectivity. Any other tested mutation at the ring of acidic residues drastically reduced infectivity: mutations D263N and D263E by ∼3 orders of magnitude and mutations E264Q and E264D by ∼5 or ∼4 orders of magnitude, respectively. The multiple mutant E146Q/D263N/E264Q in which every charge in the ring was removed was lethal; in contrast, the E146D/D263E/E264D mutant that preserved every charge but altered the stereochemistry of the 15 side chains was still infectious, as much as the single D263E mutant, and more than the single E264D mutant (Table 1, Group 4).

Comparison of the above results and those obtained by mutation of these residues to Ala (Table 1) indicates that: (i) a relatively bulky side chain (as in Glu, Asp or Gln), but not the presence of a negative charge, is required at position 146 to preserve virus infectivity; (ii) in contrast, negatively charged carboxylates at positions 263 and 264 cannot be isosterically replaced (carboxylate to amide mutations), or their position altered (Glu/Asp mutations), without drastic reductions in infectivity; both a specific side chain and a negative charge appear to be required at positions 263 (Asp) and 264 (Glu) to fully preserve infectivity.

### Molecular basis of the biological role of rings of acidic residues surrounding the capsid pores

Finally, we investigated the molecular basis for the deleterious effects of mutations at the rings of acidic residues surrounding the capsid pores. We had previously shown that a different ring of residues that closely delimit the base of each capsid pore is required to preserve MVM infectivity^66^. These residues preserve enough mechanical flexibility around the pores^67,68^ to facilitate a capsid conformational transition^69,70^ associated with through-pore externalization of biologically relevant translocation signals^56^, and are also required for other steps in the viral cycle^71^. This transition can be thermally induced in empty capsids and detected *in vitro* by following a small, but reproducible between experiments and different capsid preparations, sigmoidal variation in intrinsic fluorescence due to small changes in exposure of some Trp residues to solvent, yielding a transition temperature of ∼46 °C^69^.

**Figure 4:**
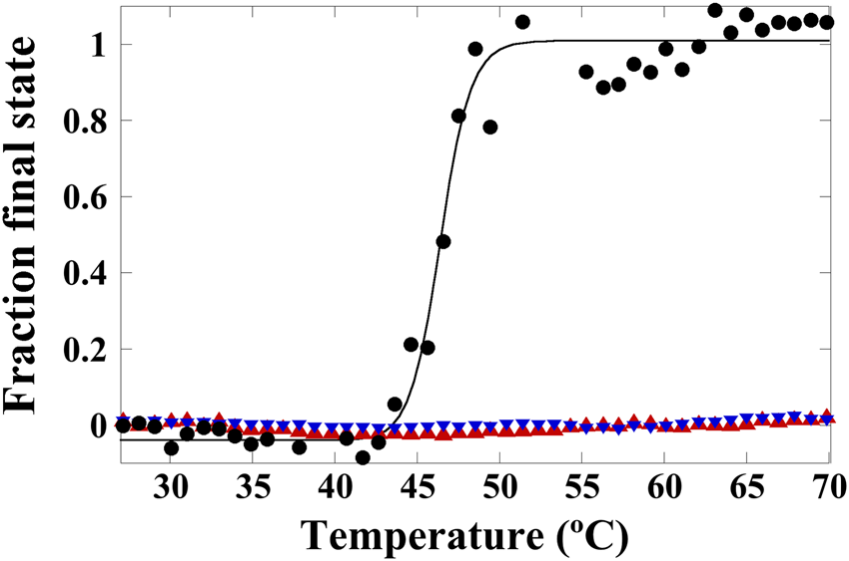
Intrinsic Trp fluorescence analysis of a heat-induced conformational rearrangement of the MVM capsid. The fraction of VP2-only capsids in the final state conformation is represented as a function of temperature. Circles, non-mutated wt control; red triangles, E146A mutant; blue inverted triangles, E264A mutant. The intrinsic Trp fluorescence of the D263A mutant as a function of temperature was determined as a part of a previous study with a different goal^66^. The *T*_*m*_ for this transition in the wt capsid varied within <1 °C in 4 independent experiments carried out for this study.

We hypothesized that, like the rings of residues delimiting the base of the pores, the rings of acidic residues surrounding the pores at a somewhat higher radius could be involved in enabling the pore-related transition. Intrinsic fluorescence analysis of E146A, D263A and E264A mutant capsids in parallel with the non-mutated control capsid revealed that any of these mutations did prevent the conformational transition from occurring (Fig. 4). To sum up, the above results indicate that the ring of acidic residues surrounding each capsid pore is required to facilitate the conformational transition associated with through-pore translocation events needed for viral infection.

## Discussion

In this study we investigated the biological role of 11 of the 28 electrically charged residues per protein subunit located at the structured inner wall of the capsid of MVM, a small ssDNA virus. In addition, effects of introducing charged groups in 5 additional positions at the inner surface of each capsid subunit were determined. The results revealed several aspects of the relationship between the presence, distribution and location of many charged residues in a virus capsid and viral function, as summarized and discussed next.

### Assembly of the MVM capsid and virus infectivity are rather tolerant to removal or introduction of electrically charged groups at the structured capsid inner Wall

As the MVM capsid does not coassemble with the viral nucleic acid, it could be thought that the weak net charge on the capsid inner surface (exactly zero if positively charged VP1 Nts and negatively charged phosphorylated residues were disregarded) could be required for efficient capsid self-assembly. In fact, in 8 out of 10 tested cases individual removal or introduction of basic side chains at the structured capsid inner wall had either no significant effect (6 cases) or only moderate influence (2 cases) on capsid assembly and virion yields. This statement holds true irrespective of the specific mutated residue, its position in the capsid inner surface, or the interactions it establishes with neighboring amino acid residues. MVM capsid assembly and virus infectivity appear to be largely tolerant to substantial changes at the structured capsid inner wall regarding net electrical charge (±60 units) and electrostatic potential distribution, that could arise through point mutations during biological evolution.

### The structured capsid inner wall of MVM may not contribute to neutralization of the electric charge of the viral ssDNA genome

Both empty capsids and virions of MVM are similarly thermostable and withstand temperatures of 70 °C for many minutes^72,73^. The observation of a close to 0, or even a (weakly) negative net charge at the inner surface of the MVM capsid (including Nts and phosphorylated amino acid residues), raises the question of how the repulsive effect of the >5000 negatively charged phosphates in the viral ssDNA is counteracted to allow efficient genome encapsidation and prevent a large destabilization of the viral particle. The excess positive net charge in the 10 VP1 Nts (+14 per Nt, +140 per capsid) could neutralize only a minor fraction of the negative charges in the ssDNA; moreover, VP1 Nts have been shown to be dispensable for genome encapsidation in MVM^74^.

Previous studies showed that encapsidated ssRNA in a nodavirus does not alter the atomic structure of the capsid but reduce its equilibrium dynamics and chemically stabilize the viral particle^75^. Likewise, capsid-bound ssDNA segments in MVM stiffened some regions of the viral particle and stabilized the virion against a heat-induced, inactivating reaction^76^ that did not involve capsid dissociation^73,77^, but led to the untimely release of the ssDNA genome^73^. Specific disruption through mutation of different (mostly nonionic) interactions between capsid inner wall and capsid-bound ssDNA segments reduced particle stiffness and lowered the activation free energy barrier of the heat-induced, virion-inactivating reaction^76^. These observations suggest that capsid-ssDNA interactions in the natural MVM virion contribute to keep the ssDNA molecule confined inside the capsid. The stabilization of the ssDNA-filled virion achieved through (essentially nonionic) capsid-ssDNA interactions could compensate, at least in part, the destabilizing effect of repulsive interactions between encapsidated ssDNA phosphates. In addition, metal ions and/or organic polycations such as spermidine, which in at least some ssRNA viruses neutralize a part of the negative charges in their genomes^35–37^, could neutralize a large fraction of the encapsidated ssDNA charges in MVM (under study).

### Removal or introduction of electrically charged groups at the capsid inner wall reduces the stability of the MVM virion against heat-induced inactivation

In 3 out of 9 tested cases, either removal or introduction of basic groups at the capsid inner wall substantially impaired the resistance of the infectious virion against heat-induced inactivation. This could possibly lead to a competitive disadvantage for these mutants compared to the wt virion in the environment, where viruses are frequently subjected to heat extremes. The three mutations that increased thermal sensitivity of the MVM virion involved capsid residues that are located close to the capsid-bound ssDNA segments (Fig. 1b). Of them, mutation R54A could be thought to debilitate an attractive ionic interaction between capsid and bound ssDNA segments, facilitating the heat-induced extracellular release of the viral nucleic acid. On the other hand, mutations, Q137K and Q255R, introduced an additional basic group that could establish attractive ionic interactions between capsid and bound ssDNA. All of the above observations together suggests, as an unproven possibility to be investigated, that the strength and distribution of electrostatic potential at the ssDNA binding sites in the MVM capsid may be conserved as a balancing act: weaker capsid-ssDNA interactions could facilitate untimely release of the genome in extracellular virions at elevated ambient temperature, whereas stronger capsid-ssDNA interactions could impair intracellular genome uncoating, leading in both cases to a selective disadvantage for the virus.

### Rings of acidic residues around pores in the MVM capsid are required for a capsid conformational transition required for viral infection

In contrast to the generally moderate or insignificant effects on capsid assembly and virion yields of removing or introducing basic groups at the capsid inner wall, removal by mutation to Ala of acidic groups at different positions in the capsid inner wall abolished virus infectivity in 5 out of 6 tested cases. Mutations D115A and D474A either drastically or significantly impaired capsid assembly, and were lethal for the virus. Truncation of the side chains of residues E146, D263, E264 that form rings of acidic residues around each capsid pore (Fig. 1c) had no significant effects on capsid assembly or virion thermal resistance, but were also lethal. More detailed mutagenic analysis revealed that the presence of a negatively charged carboxylate at positions 263 and 264 is necessary (albeit not sufficient) for preserving viral infectivity. The critical biological role of these rings of acidic residues around the capsid pores was traced to their involvement in allowing a subtle but global conformational transition of the capsid that is associated to though-pore translocation events.

The atomic structure of a variant MVM capsid with a N170A point mutation at the base of the pores that prevented that transition and was lethal for the virus has recently been determined by X-ray crystallography^68^. The structure revealed that the N170A mutation leads to a subtle but significant overall structural compaction of the viral particle and a reduction in flexibility of different structural elements delimiting the pores or located in other capsid regions; this observation is in agreement with the N170A-induced mechanical rigidification of the pore region and the capsid in general that was detected by AFM^67^. Mutation to Ala of D263 which structurally links the rings of residues delimiting the base of the pores with the ring of acidic residues at a somewhat higher radius leads also to capsid mechanical stiffening^67^. Like N170 and, perhaps, other residues at the base of the pores^66,67,71^, the rings of acidic residues could contribute, both sterically and through local electrostatic repulsions, to prevent a slight structural compaction and rigidification of the capsid and preserve a high enough conformational dynamism around the pores (under study).

## Conclusion

**Figure 5:**
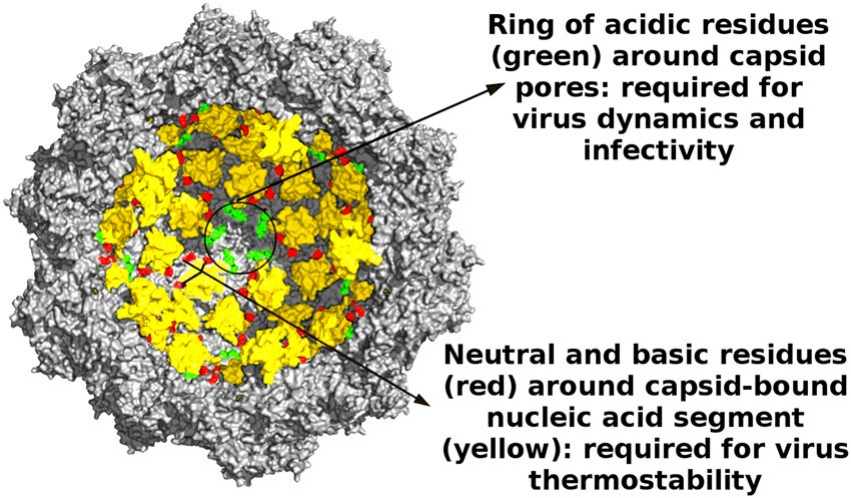
Functional roles of electrically charged residues at the inner surface of the MVM capsid. A cross-section of the atomic structure of the MVM virion^51,52^ is represented. ssDNA segments bound to the capsid inner wall are colored yellow. Residues R54, Q137 and Q255 close to the capsid-bound DNA segments are colored red. Residues E146, D263, E264 that define conspicuous rings of negatively charged carboxylates surrounding each capsid pore are colored green.

A systematic mutational analysis involving charged groups located throughout the inner wall of the capsid of a model virus, MVM, has revealed that a large fraction of these charged groups are biologically relevant (Fig. 5). Three point mutations that either increased or decreased the number of positive charges around structured capsid-bound ssDNA segments reduced the resistance of the extracellular virion against thermal inactivation. Several point mutations that either removed or changed the positions of negatively charged carboxylates in rings of acidic residues around the capsid pores were deleterious by precluding a conformational transition of the capsid associated to pore dynamics and through-pore translocation events required for viral infection. The number and distribution of charged residues at the capsid inner wall of a model virus appears to be the result of selective pressures for a compromise between different functional requirements, including virion thermostability and conformational dynamics.

## Methods

### Recombinant plasmids and mutagenesis

Recombinant plasmids pSVtk-VP1/2^78^ and pFB1-VP2^69^ respectively contain the VP1/VP2 or VP2 coding sequences in the MVMp genome. Site-directed mutagenesis on these plasmids was carried out using the QuikChange kit (Stratagene). The recombinant plasmid pTrp contains the entire genome of MVMp^79^. Mutations in this infectious clone were introduced by subcloning using the corresponding mutant pSVtk-VP1/2 as donor. pFB1-VP2 (wt and mutants) were used as donor plasmids to construct the corresponding recombinant BM-VP2 bacmids, using the Bac-to-Bac Baculovirus Expression system (Invitrogen) as indicated by the manufacturer, with minor modifications^69^. For every mutant, the presence of the introduced mutations was confirmed by DNA sequencing.

### Expression of VP2-only capsids of MVM in insect cells

Recombinant BM-VP2 bacmids (wt and mutants) were used to transfect insect cells as previously described^80^. Briefly, the transfected cells were incubated at 27 °C until nearly complete cytopathic effect (about 6 days). The recombinant baculovirus obtained was used to infect fresh cell monolayers, which were incubated at 27 °C for 3 days, and the cells were harvested and centrifuged. The washed pellet was resuspended in lysis buffer (50 mM Tris-HCl pH 8, 150 mM NaCl, 0.2% Triton X-100, 0.5 mM EDTA), and frozen at −70 °C as a source of MVM capsids.

### Purification of VP2-only capsids of MVM

MVM capsids were purified following as previously described^72^, including centrifugation through sucrose cushions and gradients. Purified capsid preparations were extensively dialyzed against phosphate-buffered saline (PBS: 8.1 mM sodium phosphate, 1.5 mM potassium phosphate, 137 mM NaCl, 2.7 mM KCl, pH = 7.5) and stored at 4 °C or −70 °C. When needed, capsids were concentrated by ultrafiltration. Purity was assessed by SDS-PAGE (the viral capsid protein, VP2 accounted for >90% of the protein detected) and electron microscopy.

### Analysis of conformational change in VP2-only capsids of MVM by spectrofluorimetry

Purified VP2-only capsids (wt and mutants) in PBS were subjected to thermal gradients, and changes in intrinsic tryptophan fluorescence were followed by spectrofluorimetry as described previously^69^. A Varian Cary Eclipse luminescence spectrophotometer equipped with a Peltier temperature control unit was used. The signature of the conformational transition being investigated was a subtle but reproducible sigmoidal variation between 40–50 °C superimposed to the otherwise linear decrease in fluorescence intensity due to thermal quenching^69^. This transition was repeatedly observed in many experiments using different preparations of wt capsids and has been thoroughly validated previously^66,67,69^. If the transition took place, it was fitted to a simple cooperative unimolecular process using equation [1] in ref.^69^, and the transition temperature *T*_*m*_ was obtained.

### Expression and assembly of VP1/VP2 capsids of MVM in mammalian cells followed by *in situ* immunofluorescence analysis

Human NB324K cells were transformed with pSVtk-VP1/2 plasmids (wt or mutants) and MVM VP expression and capsid assembly were analyzed *in situ* in immunofluorescence assays as previously described^53,55,81^ with minor modifications. The primary antibodies were: (i) a rabbit polyclonal antibody that recognizes both unassembled and assembled VP subunits of MVM (anti-VP PAb), provided by J.M. Almendral (CBMSO, Madrid) and previously validated^82^; (ii) a mouse monoclonal antibody (MAb B7) that recognizes the assembled MVM capsid only, previously described and validated^52^. Secondary antibodies were Alexa 594 and Alexa 488 (Invitrogen). For each mutant assembly efficiency was determined, and the values obtained were normalized relative to the assembly efficiency of the wt control in the same experiment, as described in footnote *c* of Table 1.

### Production of MVM virions in mammalian cells by transfection and virus titration assays

NB324K cells were transfected with pTrp plasmids (wt or mutants) as previously described, and infectious progeny virions were titrated in standard plaque-formation assays^83^. To ensure quantitative transfections, samples were first normalized for capsid protein expression in western blot assays as described^61^. For each mutant the infectious titer was determined, and the values obtained were normalized relative to the titer obtained for the wt control in the same experiment, as described in footnote *d* of Table 1.

### Thermal inactivation assays

Virus suspensions with high infectious virus titers were incubated at 70 °C (confirmar temperatura) for different amounts of time, and the titer of the remaining infectious virus was determined as described above, and the values obtained were normalized relative to the value obtained for the wt control in the same experiment as described in footnote *e* of Table 1.

### Molecular graphics and structural analyses

The PDB coordinates of MVMp empty capsid (1Z14) and MVMi virion (1Z1C) atomic structures^52^, and the programs WHATIF^84^, RasMol^85^ and Pymol (W.L. DeLano, http://www.pymol.org) were used for molecular graphics and analysis of viral structures.

### Data availability

All data generated or analysed during this study are included in this published article.
